# Genome mining of amylases and amylase inhibitors from Streptomyces

**DOI:** 10.1099/mgen.0.001747

**Published:** 2026-06-05

**Authors:** Andreas C. Lawaetz, Alexa Gannon, Paul A. Hoskisson

**Affiliations:** 1 Strathclyde Institute of Pharmacy and Biomedical Sciences, University of Strathclyde, 161 Cathedral Street, Glasgow, G4 0RE, UK

**Keywords:** acarbose, amylase, pullulanase, starch, *Streptomyces*, tendamistat

## Abstract

Most antibiotics are derived from *Streptomyces* bacteria and produced industrially via fermentations relying on food-grade feedstocks, which have a substantial environmental impact. Efficient utilization of starch-rich organic wastes, such as bread and potato waste, as alternative feedstocks, remains limited by incomplete knowledge of starch metabolism in *Streptomyces*. Here, a genus-wide analysis of 295 *Streptomyces* strains was performed, identifying 645 amylases grouped into pullulanases, *α*-1,4-amylases and cyclomaltodextrin-like amylases, alongside biosynthetic gene clusters encoding amylase inhibitors such as acarbose and tendamistat. Structural and sequence analyses revealed that inhibitor resistance may arise from subtle modifications in the *α*-amylase catalytic pocket. This comprehensive analysis of amylases and inhibitors provides a foundation for engineering inhibitor-resistant enzymes tailored to diverse starch substrates, facilitating the development of sustainable, starch-based *Streptomyces* fermentations.

**Impact Statement**

Pharmaceutical manufacturing contributes substantially to global carbon emissions, with feedstocks used in natural product fermentations being a major factor. Replacing conventional feedstocks with organic waste streams, such as starch-rich bread or potato waste, could reduce the environmental impact, but two key challenges remain: production strains may lack the enzymes required to efficiently degrade alternative substrates, and nutrient changes may trigger carbon catabolite repression, reducing product yields. While previous studies have linked carbohydrate-active enzymes with biosynthetic gene clusters, no comprehensive analysis has focused specifically on starch degradation in *Streptomyces*. To fill this gap, a genus-wide analysis of amylases and amylase inhibitors across 295 *Streptomyces* genomes was carried out. This created an atlas of 645 amylases, which may serve as the foundation for the targeted selection of enzymes optimized for catabolizing organic waste from various sources, thereby improving the sustainability of industrial natural product production, such as antibiotics.

## Data Summary

Scripts used along with supplementary files and figures can be accessed through GitHub at https://github.com/ALawaetz/Amylases_and_amylase_inhibitors_in_Streptomycetes.

## Introduction

The majority of antibiotics and other bioactive natural products are generated by complex biosynthetic pathways found naturally in *Streptomyces* bacteria and are produced industrially in large-scale fermentations [1]. Pharmaceutical manufacturing has a substantial impact on the environment and accounts for 5% of the UK’s carbon footprint with a large contribution coming from the carbon-intensive processed sugars and lipids currently used as feedstocks in antibiotic production systems [2]. One strategy to enhance the sustainability of pharmaceutical manufacturing is to replace current feedstocks with organic waste products. Globally, ~1.3 billion tons of food waste is produced each year, representing a cheap and sustainable energy source that could become part of a circular bioeconomy instead of ending up in landfills [3,4]. Bread waste, for example, is comprised of 50–70% starch, which could potentially be fed into antibiotic fermentations [4,5].

Changing feedstocks for bioactive natural products, such as antibiotics in production strains, however, is not straightforward, as primary metabolism is intimately linked to biosynthesis, which is in turn subject to carbon catabolite repression [6]. To rationally engineer cells to metabolize desired carbon sources while maintaining high antibiotic titres, a thorough understanding of metabolic networks is required. Hence, substantial efforts have gone into correlating the repertoire of biosynthetic gene clusters (BGCs) with that of carbohydrate-active enzymes (CAZymes) in many *Streptomyces* genomes [7,9]. While CAZyme analyses use resources such as the Carbohydrate-Active Enzyme database [10] and annotation tools like dbCAN [11] and provide valuable insights into the metabolic breadth of organisms, they are limited by the level of functional detail they can offer. Many CAZyme categories are broad and encompass proteins with diverse enzymatic activities. For example, the glycoside hydrolase category 13 (GH13) family contains enzymes with activities as diverse as amylases, sucrases and trehalose synthases. Whilst efforts are underway to further subdivide CAZyme families into more homogeneous, functionally coherent groups [10], these refinements remain incomplete. As a result, previous efforts of genome mining constricted to CAZyme-based approaches do not resolve specific carbohydrate utilization pathways. Therefore, to bioinformatically assess the capacity of streptomycetes to metabolize starch specifically, further characterization beyond standard CAZyme annotation is required.

Starch is a naturally occurring polymer and many bacteria, including species of *Streptomyces*, encode and secrete amylases, which enable them to break down and catabolize starch [5]. Starch consists of long chains of glucose residues covalently linked through *α*-1,4-glycosidic bonds and may further be branched through *α*-1,6 linkages (amylose and amylopectin, respectively) [5]. In the natural microenvironments of streptomycetes, other starch-like polysaccharides may be encountered, including pullulan (composed of maltotriose units linked by *α*-1,6 bonds) and cyclomaltodextrins (cyclic oligomers of *α*-1,4-linked glucose residues). Concordantly, *Streptomyces* genomes have been annotated as encoding amylases, pullulanases and cyclomaltodextrinases [5]. Starch is among the most abundant and energy-dense polymers in the environment [5], and fierce competition between microbes to access this resource is likely. Unsurprisingly, species of *Streptomyces* synthesize amylase inhibitors, including carbohydrate-based compounds like acarbose, acarviostatin and trestatin and small peptide inhibitors like tendamistat, Gaim and Scaim [12,16]. While amylase inhibitors and inhibitor-resistant amylases have been characterized in some species, their prevalence and correlation with the repertoire of amylases across the *Streptomyces* genus have not been systematically assessed. It remains unclear whether inhibitor production and inhibitor-resistant amylases represent isolated traits or whether they form part of a broader ecological strategy linked to starch utilization. Addressing this gap is important for the development of sustainable fermentation processes based on starch-rich waste streams, where efficient substrate utilization is critical. The ability of *Streptomyces* strains to metabolize such feedstocks optimally may depend not only on the presence of amylases but on their exact substrate specificities and susceptibility to inhibition. Resolving these factors is, therefore, essential for the rational selection or engineering of strains optimized for the sustainable manufacturing of specialized metabolites.

The saprophytic nature and abundance of starch in the natural environment of *Streptomyces* [5], coupled with the ability to produce numerous specialized metabolites, led to the hypothesis that starch utilization is a common trait in streptomycetes, which is intricately linked to the production of amylase inhibitors and the evolution of inhibitor-resistant amylases. To test this, bioinformatic resources, including dbCAN [17], SignalP [18], hmmer [19] and GATOR [20], were combined to assess the distribution of amylases and amylase inhibitors across 295 *Streptomyces* genomes [21], providing a broad representation of the starch catabolic diversity of the genus. It was found that pullulanases and *α*-1,4-acting amylases are almost ubiquitous, whereas only a subset of strains encodes a third, cyclomaltodextrin-like amylase. The total number of amylases per genome ranged from zero to six, and there was a positive correlation between strains encoding higher numbers of amylases and those encoding amylase inhibitors. BGCs encoding amylase inhibitors were typically found alongside multiple amylases, including variants with amino acid motifs known to provide resistance to inhibition [16] as well as putative resistance variants.

## Methods

### Phylogenetic analysis of amylases in *Streptomyces*

A database of 295 *Streptomyces* genomes [21] (accession numbers found in File S6) was used for the analysis and the protein fasta files for each genome were downloaded with the National Center for Biotechnology Information (NCBI) datasets command-line tools (script: File S10, available in the online Supplementary Material). In brief, the *Streptomyces* genomes dataset [21] was selected as follows: 873 publicly available *Streptomyces* genomes containing single-copy variants of all 6 MLST markers used in the pubMLST *Streptomyces* scheme [22] were analysed using a minimum spanning tree approach [21]. This analysis resolved the genomes into 295 sequence type (ST) groups. A single representative genome from each ST group was then selected for downstream phylogenomic analysis, which was conducted using a concatenated alignment of 137 single-copy orthologue genes [21].

Amylases were found by using dbCAN (version: 5.1.2) [11] with the option CAZyme_annotation (script: File S7). Amylases belong to the CAZyme family GH13, so results were subsequently filtered for GH13 proteins where this was the recommended result (i.e. agreement between HMMER and DIAMOND). Proteins whose recommended result included multiple CAZyme groups, e.g. GH13 and/or CBM20, were also included in the analysis. Results that included subgroups, e.g. GH13_32, were included in the analysis simply as GH13 (script: File S8). SignalP (version: 6.0) [18] was then used to select only proteins with secretion signals (script: File S9), yielding a total of 645 amylases. A multiple sequence alignment of all amylases was carried out using Mafft (version: 7.525) [23] (script: File S11), and their phylogenetic relationship was determined using RAxML-NG (version: 1.2.2) [24] with 100 bootstrap replicates (script: File S12). A circular tree rooted at the midpoint was drawn from the resulting output using R (script: File S13). Domain analysis was carried out using NLM’s conserved domain database [25] using representative proteins from each clade. Representative proteins were defined as the proteins most similar to the consensus sequence in each clade (script: File S22). These were WP_062143504.1 (Clade 1), WP_218063397.1 (Clade 2) and WP_150491328.1 (Clade 3).

### Amylase inhibitors

Previously characterized BGCs encoding amylase inhibitors acarbose [16], acarviostatin [13] and trestatin [16] were used to search *Streptomyces* genomes using GATOR-GC (version: 0.9.0) [20] (script: File S14). GATOR-GC takes two lists of genes as input: a list of required genes and a list of optional genes. Required genes to search for acarbose BGC were the core genes required for aminocyclitol synthesis: *gac*(A, V, W, X, Y, U, S, R, K, I, Q, C, J, M, O). The homologous genes in *Streptomyces coelicoflavus* ZG0656 and *Streptomyces dimorphogenes* ATCC 31484 were used to search for acarviostatin and trestatin BGCs, respectively. A combined list of optional genes was used to search all clusters. This included all amylases and proteinaceous amylase inhibitors from the characterized BGCs in *Streptomyces glaucescens* [15,16], *S. coelicoflavus* ZG0656 [13] and *S. dimorphogenes* ATCC 31484 [16], all 645 amylases identified in this study and tendamistat from *Streptomyces tendae* (M28478.1). A genus-wide search for tendamistat-like peptides was carried out by building a HMMER profile (HMMER version: 3.4) from a tendamistat seed alignment (smart domain smart00783 from the NLM’s conserved domain database) and then using hmmsearch (script: Files S15) with an *E*-value cutoff of 10^−10^ to find occurrences across the *Streptomyces* genus. Occurrences of amylases and amylase inhibitors were plotted using R around a circular tree rooted at the midpoint, displaying the evolutionary relationship between 295 *Streptomyces* genomes based on whole-genome phylogeny [21] (script: File S16).

### Multiple sequence alignment

Multiple sequence alignments of each identified clade of amylases were carried out with Mafft (version 7.525) [23] using the seed option (seed alignment from conserved α-amylase domain cd00551) (File S11). Aligned sequences were visualized with Jalview (version: 2.11.5.1) [26].

### Statistical analysis

The distribution of the number of amylases per genome was tested using scipy.stats shapiro package and found not to be normally distributed. A Mann–Whitney *U* test was hence applied to test whether the distributions of the number of amylases in genomes with and without acarbose BGC were significantly different from each other. All genomes carried either one or zero cyclomaltodextrin-like amylases and one or zero tendamistat genes, and with two binary variables, a chi-square test was used to test the correlation between the two. To test for enrichment of aligned amino acids in amylases found in acarbose BGCs, a Fisher’s exact test was applied and corrected for multiple testing using FDR (Benjamini–Hochberg) and Bonferroni. A *P*-value of 0.05 was used as a cutoff for significance. All scripts used for statistical analysis can be found in Files S17 and S18.

### Protein structures and ligand binding modelling

AlphaFold 3 [27] was used to model the 3-dimensional structure of proteins. CB-DOCK2 [28] and ClusPro 2.0 [29] were used to predict the binding of acarbose (PubChem CID 9811704) and Gaim to amylases, respectively. Default parameters were used in all cases. For simplicity, the modelling of binding interactions between Gaim and pullulanases was done using the *α*-amylase domains only. As such, it cannot be excluded that amylase inhibitor resistance may be achieved through interdomain interactions obstructing Gaim binding. Open source PyMOL was installed using conda and used to visualize and annotate proteins and protein–ligand interactions (script: Files S19–S21).

## Results

**Fig. 1.**
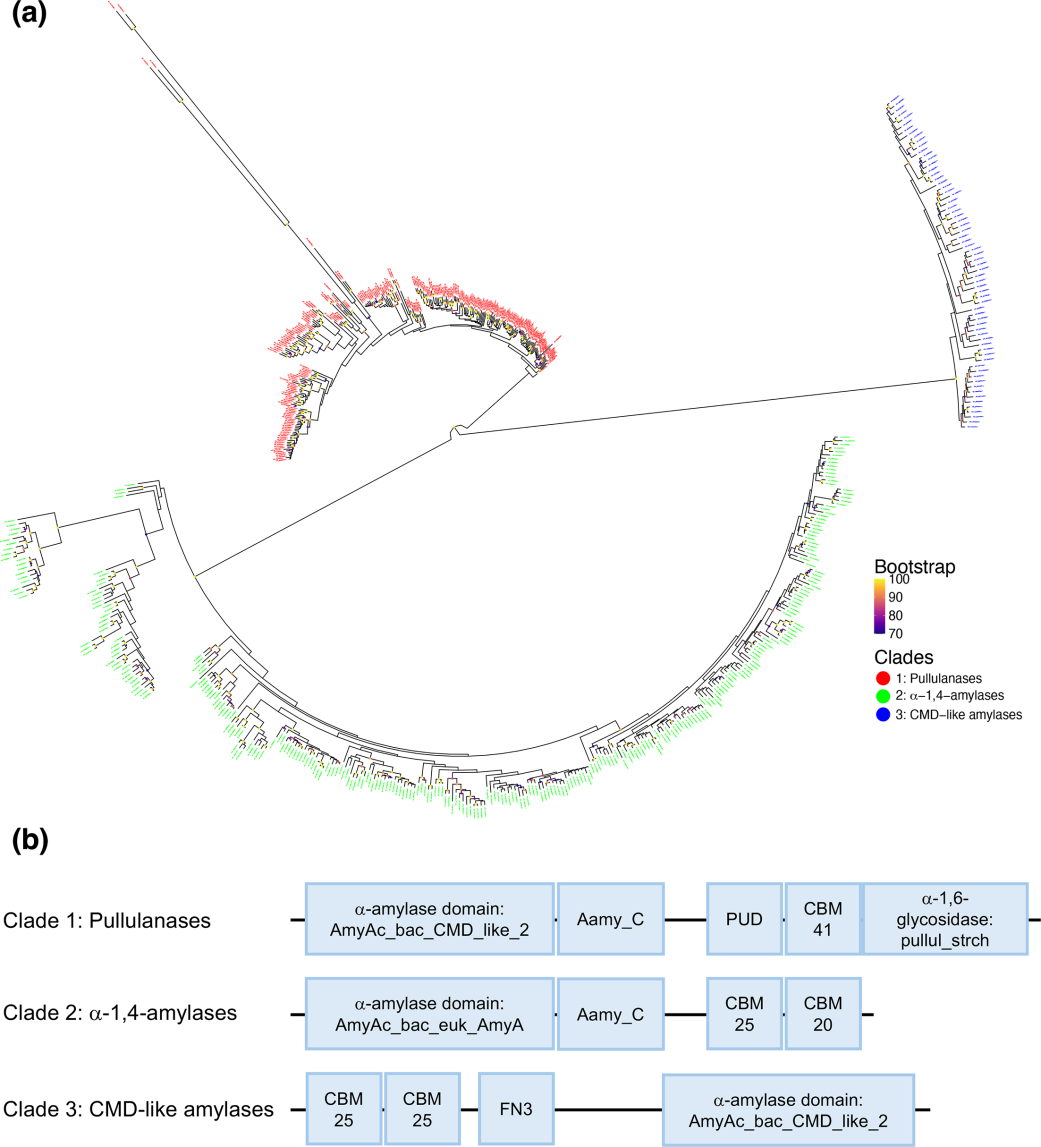
Streptomycetes possess three types of amylases. A total of 645 amylases were found across 295 *Streptomyces* genomes. Phylogenetic analysis showed that amylases in streptomycetes cluster into three major clades. Branch support values (bootstrap) are shown as Felsenstein’s bootstrap proportions scores, with coloured circles indicating values ≥70% at internal nodes (a). Conserved domain analysis of representative proteins from each clade found that clade 1 consisted of pullulanases, clade 2 of *α*-1,4-acting amylases and clade 3 of cyclomaltodextrin (CMD)-like amylases (b). AmyAc_bac_CMD_like_2, *α*-amylase catalytic domain found in bacterial cyclomaltodextrinases; Aamy_C, maltogenic C-terminal amylase domain; PUD, pullulanase-associated domain; CBM, carbohydrate-binding module; pullul_strch, pullulanase type *α*-1,6-glycosidase domain; AmyAc_bac_euk_AmyA, *α*-1,4 amylase catalytic domain found in bacterial and eukaryotic *α*-amylases; FN3, fibronectin type 3 domain. Domains not drawn to scale.

**Fig. 2.**
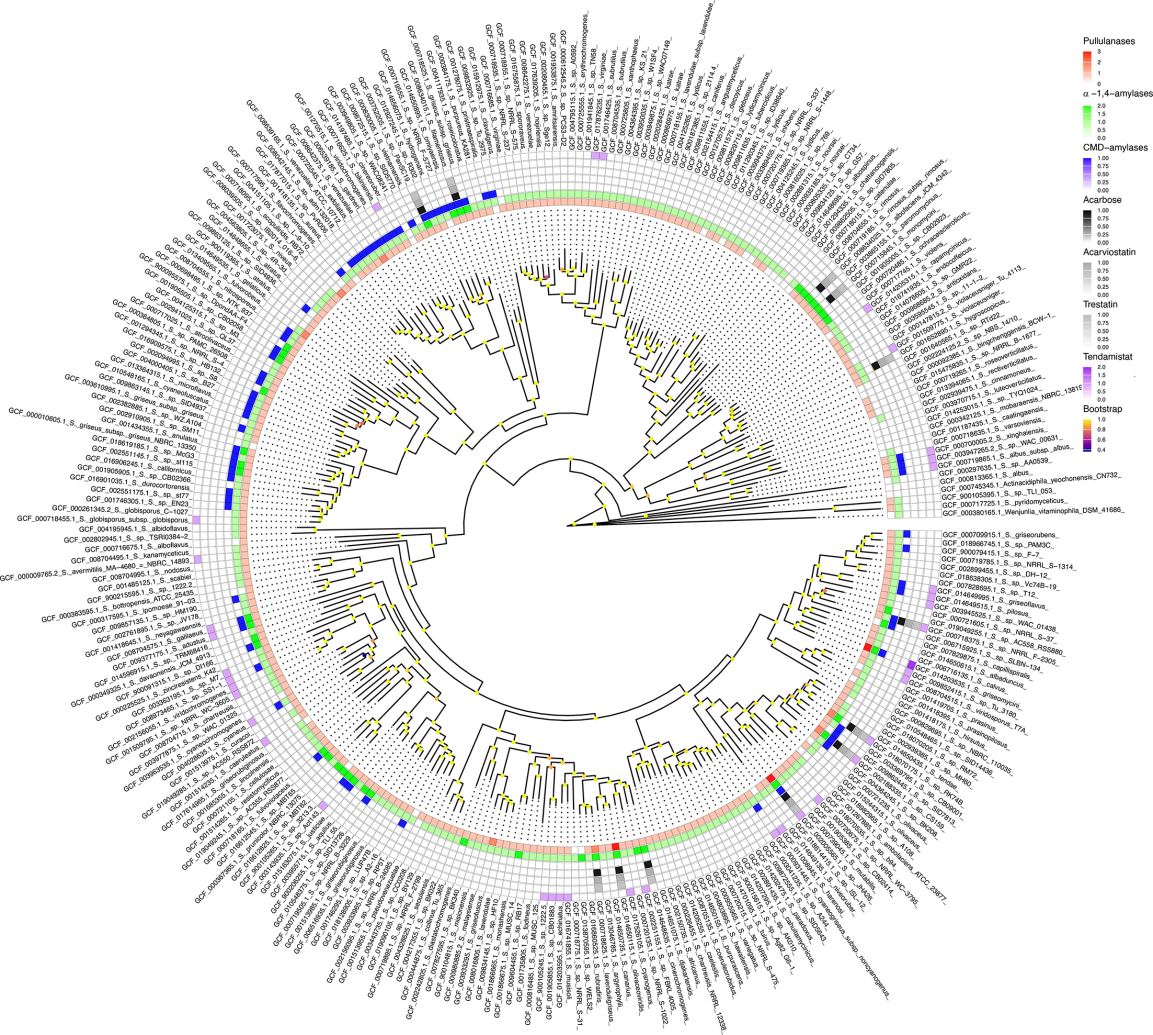
Amylases frequently co-occur with amylase inhibitors in *Streptomyces* genomes. The evolutionary relationship between 295 *Streptomyces* species was inferred from whole-genome phylogeny, and the prevalence of amylases and amylase inhibitors is visualized using a colour scale (white indicates absence; darker colours indicate higher prevalence). Annotations shown in the legend (right panel), listed from top to bottom, correspond to the concentric circles around the phylogenetic tree from the innermost to the outermost. Pullulanases (innermost, red) and *α*-1,4-amylases (green) are found almost ubiquitously throughout the *Streptomyces* genus, whereas only a subset of species encodes cyclomaltodextrin-like amylases (blue). Genomes with higher numbers of amylases frequently coincide with those encoding amylase inhibitors, including acarbose (black), acarviostatin (dark grey), trestatin (grey) and tendamistat (purple). Branch support values (bootstrap), computed as transfer bootstrap expectation scores, are represented by coloured circles at internal nodes.

To assess the prevalence of amylases in *Streptomyces* species, dbCAN [11] was used to search 295 genomes, broadly encompassing the genetic diversity of the genus [21], for proteins belonging to the CAZyme family GH13 [10]. Amylases are secreted enzymes that hydrolyse starch in the extracellular environment (Janec̆ek, 1997). The presence of a signal peptide, therefore, allows them to be distinguished from other non-secreted GH13 family members, such as enzymes involved in trehalose and glycogen metabolism [30,32]. A total of 4,173 GH13 proteins were analysed with SignalP [18], which identified 645 enzymes with secretion signal peptides. Conserved domain analysis [25] showed that all 645 contained either an amylase domain, a pullulanase domain, or both (File S23). An additional 24 GH13 proteins contained amylase domains but lacked identifiable signal peptides and were hence omitted from further analysis. Phylogenetic analysis carried out on the 645 identified amylases revealed three discrete clades (Fig. 1a). Analysis of conserved domains found that the three clades constituted pullulanases (clade 1), *α*-1,4-amylases (clade 2) and cyclomaltodextrin-like amylases (clade 3, Fig. 1b). To assess the distribution of each type of amylase across the *Streptomyces* genus, the presence of each was mapped to each genome using a phylogenetic tree displaying the evolutionary relationship between all 295 genomes (Fig. 2). It was found that most strains encoded pullulanases and *α*-1,4-amylases (87% and 89%, respectively), whereas only 22% of strains had a cyclomaltodextrin-like amylase in their genome. The total number of amylases per genome varied between zero and six.

**Fig. 3.**
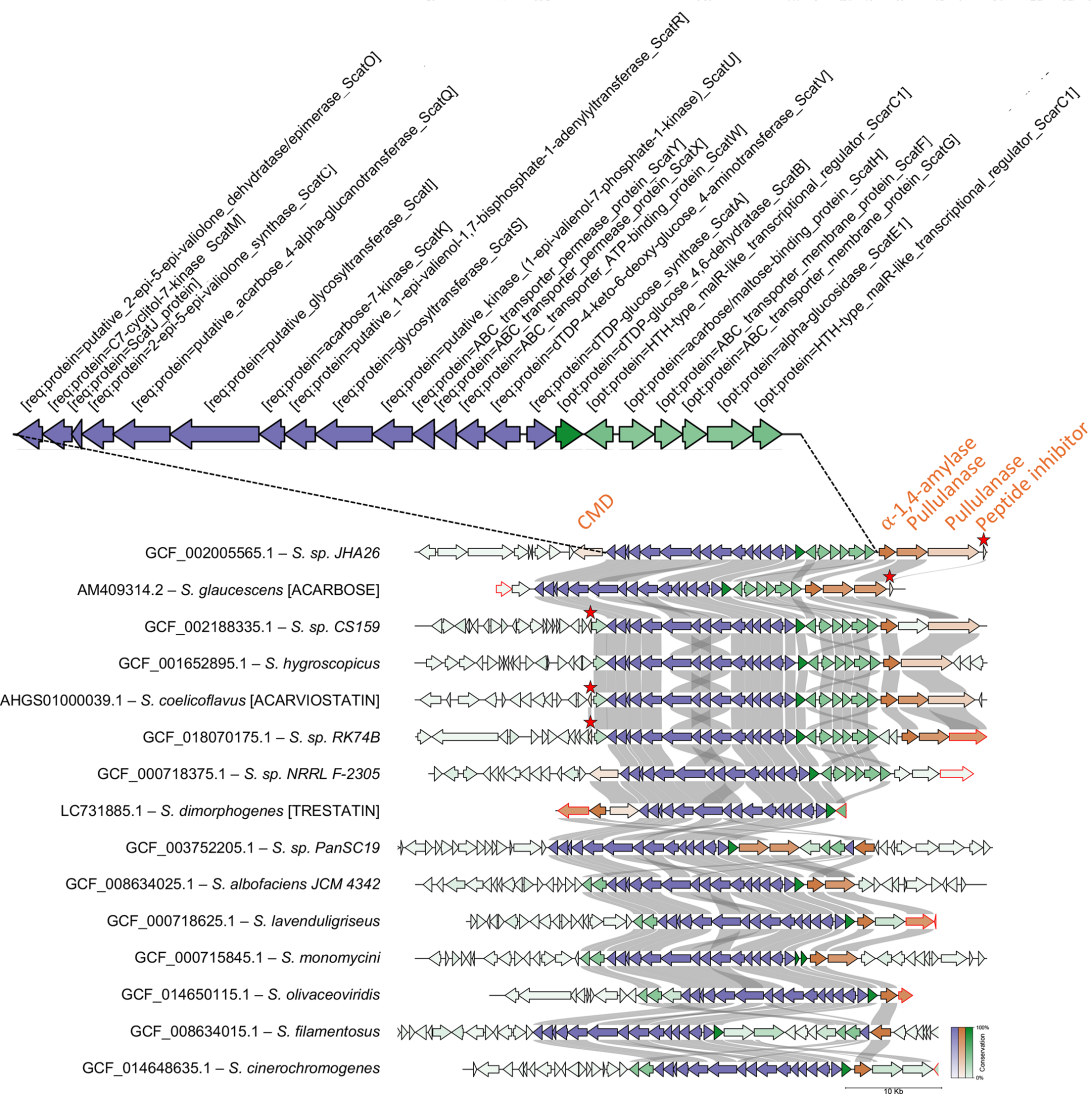
Acarbose-like BGCs possess between one and four amylases in their gene neighbourhoods. GATOR identified 15 genomes (12 from the representative list of 295 genomes plus the acarviostatin, acarbose and trestatin BGCs already described in literature). Core genes required for the synthesis of aminocyclitol are shown in blue, amylases are shown in orange and other genes in green. Tendamistat-like peptide inhibitors are shown in orange and marked with a red star. Gene-level conservation between clusters is shown by colour intensity (more intense colour for a higher level of conservation). Genes with red edges indicate contig edges. CMD, cyclomaltodextrinase.

**Fig. 4.**
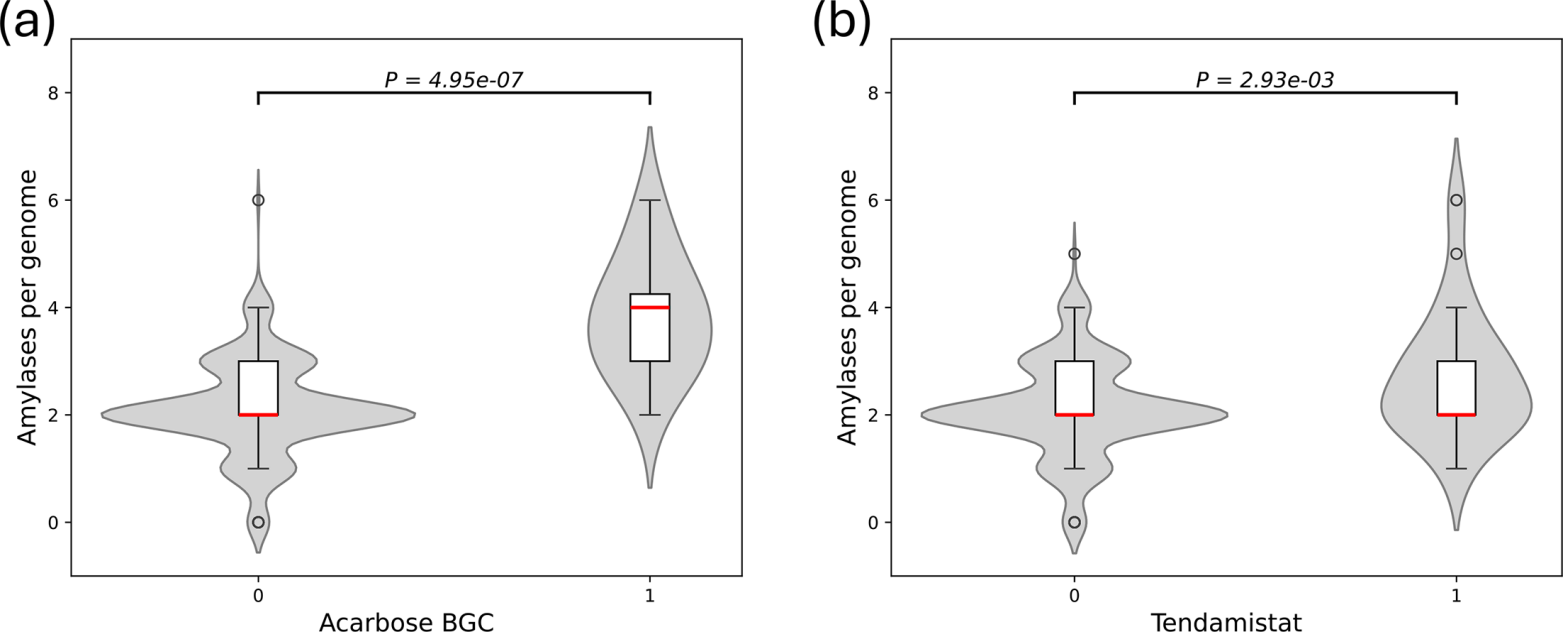
*Streptomyces* species encoding amylase inhibitors possess more amylase genes. Violin-boxplots showing significant correlations between higher numbers of amylase per genome and genomes encoding acarbose BGCs (a) (*r*=0.78; *P*=4.95×10^−7^) or tendamistat (b) (*r*=0.27; *P*=2.93×10^−3^).

Previous studies have reported BGCs encoding amylase inhibitory molecules acarbose, acarviostatin and trestatin located adjacent to amylase gene arrays [13,15, 16]. To test if strains encoding amylase inhibitors generally possess more amylase genes, an hmmsearch [19] with a tendamistat HMM profile was used to analyse the genomes and found 38 species encoding tendamistat homologs. Using GATOR [20], 12 species were identified having BGCs encoding acarbose, acarviostatin or trestatin (Figs2  3). These clusters were typically located adjacent to multiple amylase genes (numbers ranging from one to four, Fig. 3). Acarbose, acarviostatin and trestatin are all pseudooligosaccharides, but they vary in the number of C_7_N-aminocyclitol units they contain [16]. It was found that all acarbose-like gene clusters harboured a common set of genes required for C_7_N-aminocyclitol synthesis (see the ‘Methods’ section), but differed in the domain architecture of a pseudoglycosyltransferase responsible for the non-glycosidic C-N bonding in

pseudooligosaccharides [33]. Further variation was seen in their neighbouring genes including homologs of the acarbose importer AcbFGH [34,35], which was absent in some clusters (Fig. 3). A Mann–Whitney *U* test revealed that genomes encoding acarbose BGCs had significantly higher numbers of amylases and a large effect size compared to those without such clusters (*r*=0.78; *P*=4.95×10^−7^) (Fig. 4a). A similar analysis found a significant correlation between higher number of amylases and genomes encoding tendamistat but with a smaller effect size (*r*=0.27; *P*=2.93×10^−3^) (Fig. 4b). A chi-square test further revealed a positive correlation between cyclomaltodextrin-like amylases and tendamistat with a small effect size (phi=0.16; *P*=0.009). No significant difference in genome size was found between strains encoding acarbose (*P*=0.40) or tendamistat (*P*=0.29) and those that did not.

Previous studies in *S. glaucescens* and *S. dimorphogenes* pairing *in silico* analysis with mutational screening identified a catalytic pocket in pullulanase domain containing *α*-amylases where substitution of a histidine with alanine or asparagine was sufficient to confer resistance to acarbose [16]. To evaluate whether this might be a general feature of amylases associated with acarbose-like BGCs, a multiple sequence alignment (MSA) was constructed (File S5) and found seven amylases across six genomes with acarbose-like BGCs having a histidine to asparagine substitution at this catalytic site. Although 11 genomes lacking such BGCs also encoded a pullulanase with the histidine to asparagine substitution (Fig. S5), a two-sided Fisher’s exact test found it 30-fold enriched in species harbouring acarbose-like clusters (*P*=5.56×10^−6^). The substitution was only found in pullulanase domain-containing amylases. Interestingly, not all species encoding acarbose had an amylase with the described histidine to asparagine substitution (Fig. S1), suggesting that acarbose resistance may be achieved through other mechanisms.

**Fig. 5.**
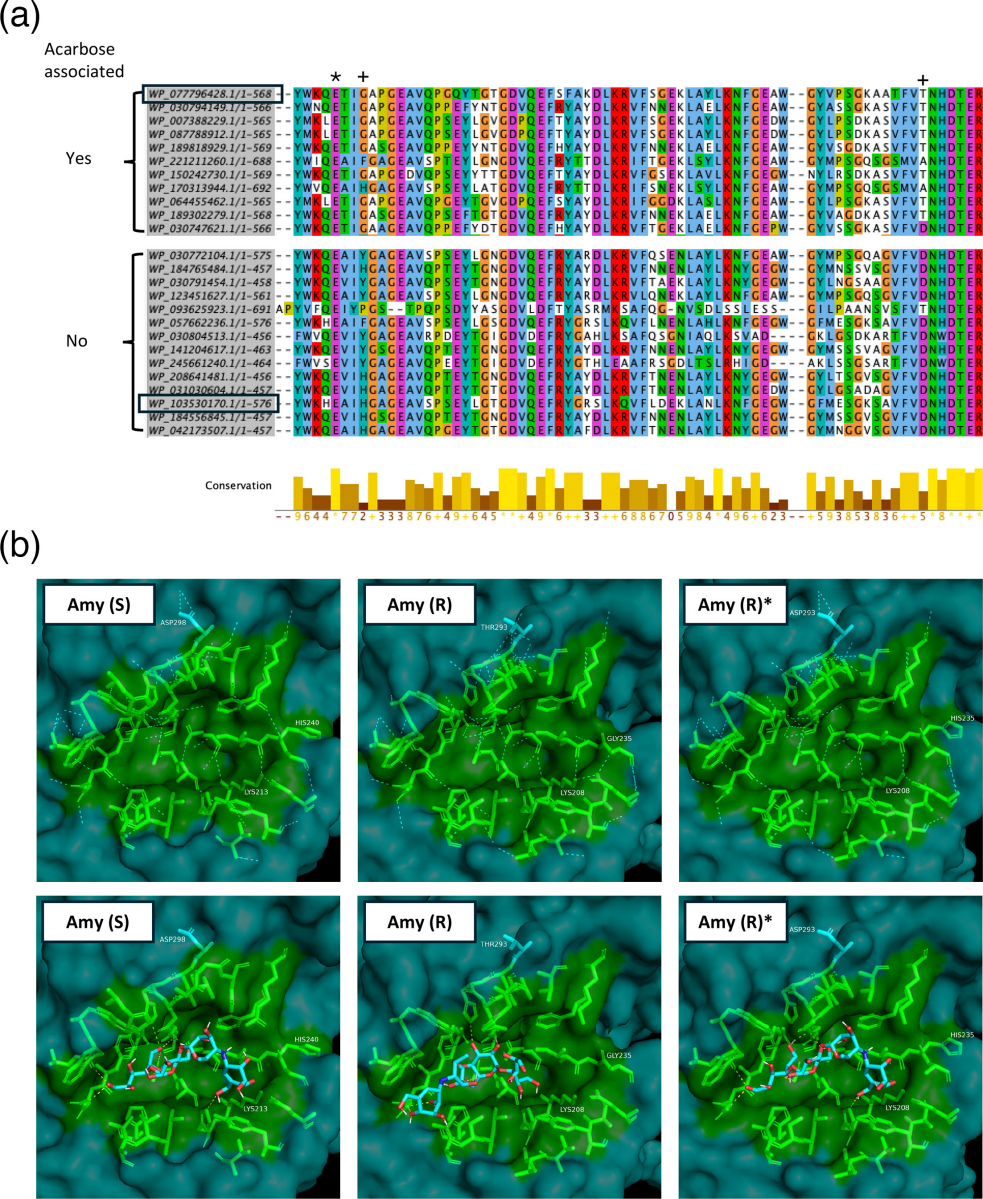
Predicted binding sites of acarbose differ between amylases that are located in acarbose BGCs and amylases that are not. MSA shows an enrichment of GLY235 and THR293 (+) in alpha-1,4-amylases located in acarbose BGCs. Both residues are found within the conserved catalytic pocket close to the catalytic residue glutamate (*) (a). The catalytic pockets (residues highlighted in green) are shown for a putatively acarbose-sensitive amylase [Amy (S) – accession: WP_103530170] found in a genome without acarbose and a putatively resistant amylase [Amy (R) – accession: WP_077796428] found within an acarbose BGC [proteins marked by black squares in (a)]. Amy (R)* shows the structure of Amy (R), where GLY235 and THR293 have been converted to HIS and ASP, identical to the residues found at these positions in Amy (S). Hydrogen bonds between residues within the catalytic pocket are shown with dashed cyan lines, and hydrogen bonds between acarbose and the proteins are shown with dashed yellow lines (bottom panel). LYS213/208, previously described as involved in inferring acarbose resistance, is highlighted (b).

To find possible acarbose-resistant amylases, an MSA was constructed for each clade of amylases (pullulanases, *α*-1,4-amylases and cyclomaltodextrin-like amylases), identifying numerous amino acid motifs significantly enriched in species harbouring acarbose-like BGCs (Files S2–S4). In acarbose BGC-associated *α*-1,4-amylases, it was found that they possess several amino acid changes including a H/Y to G and a D to T substitution (Fig. 5a). Using AlphaFold3 [27] to model the structures of an acarbose-associated *α*-1,4-amylase (WP_077796428.1) and a non-acarbose-associated amylase from the same clade (WP_103530170.1), a noticeable difference in the architecture of the catalytic pocket [16,36,38] was found between the two groups (Fig. 5b). Importantly, using CB-DOCK2 [28] to model the binding of acarbose, it was found that the inhibitor is predicted to bind both amylases but in different orientations (Fig. 5b). To assess, *in silico*, the effect of the H235G and D293T substitutions in the acarbose-associated amylase, the amino acids were reverted to H and D, respectively, and it was found that this alone was enough to widen the catalytic pocket and change the binding configuration of acarbose similar to that predicted for the non-acarbose-associated amylase (Fig. 5b). A similar conversion was observed by making the reciprocal substitution in the non-acarbose-associated amylase (Fig. S2). Importantly, modelling of an *α*-1,4-amylase (WP_150242730) from an acarbose BGC without a pullulanase having the H to N acarbose-resistance-substitution [16] also displayed an inhibitor-resistant acarbose binding phenotype that could be reversed through a single G238H substitution (Fig. S5). It was hypothesized that reorienting the binding of acarbose within the catalytic pocket might be a general way of acquiring acarbose resistance, and, therefore, the binding of acarbose to an acarbose-associated pullulanase (WP_064455463) was modelled. It was found that acarbose was predicted to bind similarly (Fig. S3) to that determined for the putatively acarbose-resistant *α*-1,4-amylase, with the imino moiety of acarbose oriented away from the region of the binding pocket known to be instrumental in establishing acarbose resistance [16]. Notably, these predictions are consistent with previously reported structural modelling and docking analyses of acarbose and pullulanases [16]. The asparagine to histidine conversion appears to result in the reversion of this group of pullulanases from an inhibitor-resistant to an inhibitor-sensitive conformation [16], and the modelling predicts a reorientation of acarbose binding (Fig. S3). A lysine residue directly adjacent to this site is conserved in both pullulanases and *α*-1,4- amylases (File S5), and as described previously, the orientation of this residue’s side chain varies considerably between resistant and sensitive amylases [16]. Similar observations were found in *α*-1,4-amylases (Fig. S4), likely attributable to altered hydrogen bonding networks in the catalytic pocket (Fig. 5).

**Fig. 6.**
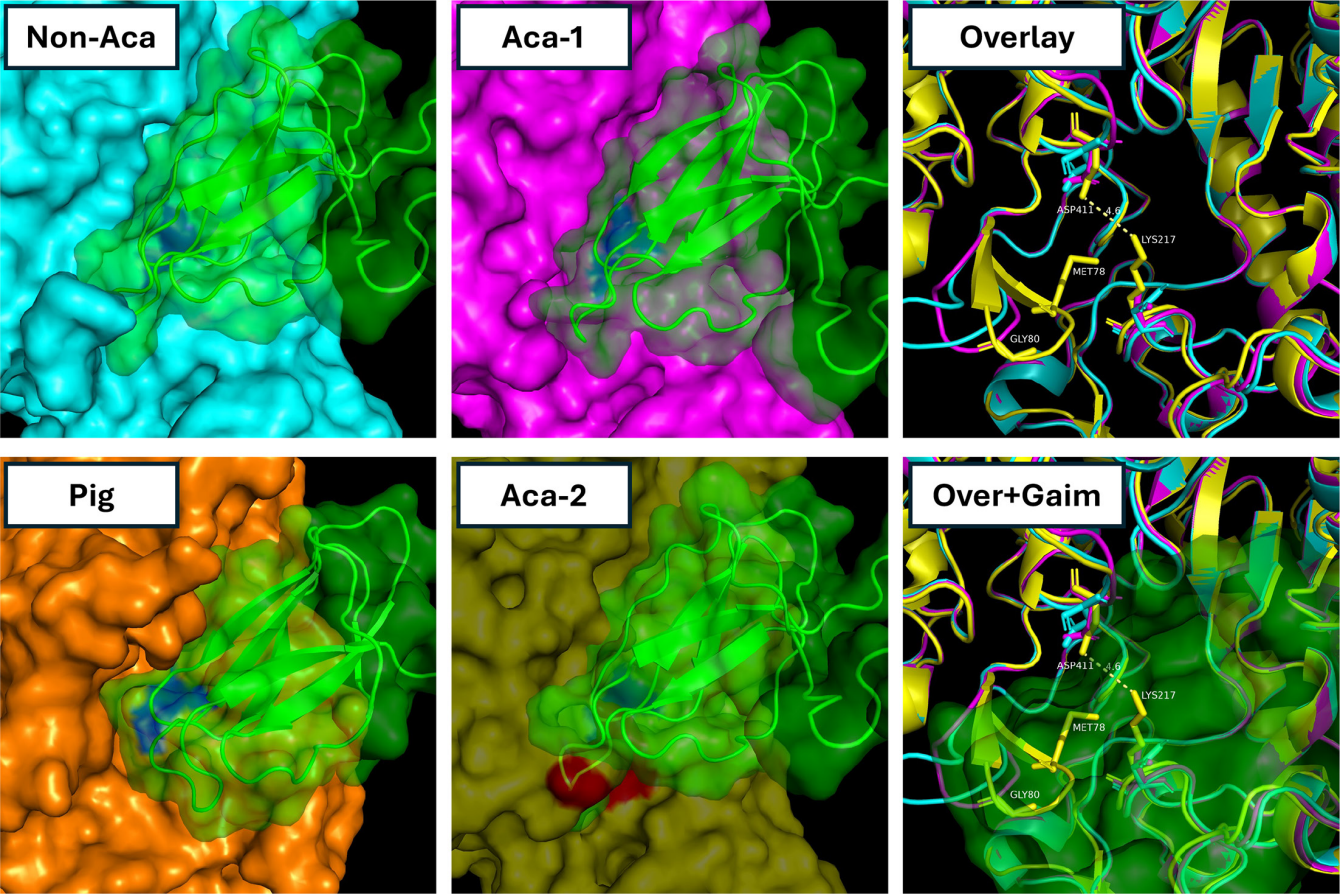
Structure predictions and protein-protein docking analyses suggest obstructed binding of proteinaceous amylase inhibitor Gaim to an acarbose-associated pullulanase from *Streptomyces* sp. JHA26. Gaim is predicted to bind two pullulanases from *Streptomyces* sp. JHA26: one encoded within the acarbose BGC [Aca-1 (magenta); WP_077796430.1] and one located outside the BGC [non-Aca (cyan); WP_077796080.1]. Gaim (green) encoded within the acarbose BGC is predicted to bind in the starch binding pocket containing the catalytic aspartate and glutamate residues (blue), like the binding of tendamistat (green) to porcine pancreatic amylase (orange, Pig). Alignment of the second acarbose BGC-encoded pullulanase [Aca-2 (transparent yellow); WP_077796429.1] with the Gaim-bound non-acarbose-associated pullulanase reveals three protruding amino acid residues (red) that are predicted to obstruct Gaim binding. An overlay (Overlay) of the three *Streptomyces* amylases highlights these residues (MET78, GLY80 and LYS217), including weak electrostatic interactions between ASP411 and LYS217, which creates a closed pocket confirmation that sterically clashes with Gaim binding (Over+Gaim).

The acarbose and acarviostatin BGCs of *S. glaucescens* and *S. coelicoflavus* encode in addition to pseudooligosaccharides, also small proteinaceous amylase inhibitors, Gaim and Scaim [13,16]. These peptides contain the conserved amylase inhibitor domain (smart00783) also found in tendamistat. Tendamistat is known to inhibit eukaryotic amylases [39,40]. Little is known, however, about its activity against prokaryotic amylases, and to the authors’ knowledge, no studies have investigated potential resistance mechanisms to tendamistat or tendamistat-like peptides. GATOR identified three additional *Streptomyces* species that harbour acarbose BGCs encoding small peptides with amylase inhibitor domains (Fig. 3). To assess potential inhibition of *Streptomyces* amylases, interactions between Gaim and three pullulanases from *Streptomyces* sp*.* JHA26 (GCF_002005565.1)*,* two encoded within the acarbose BGC and one outside the cluster were modelled using ClusPro [29]. Both BGC-associated pullulanases contain the histidine to asparagine substitution known to confer resistance to acarbose [16], while the non-BGC-associated pullulanase does not. Gaim was predicted to bind in the α-amylase catalytic pocket of the non-BGC-associated pullulanase as well as one of the two BGC-associated pullulanases, resembling the resolved interaction between tendamistat and porcine pancreatic amylase (Fig. 6 [40]). In contrast, no comparable binding was predicted for the second BGC-associated pullulanase. This appears to be due to an obstructed binding pocket formed by protruding side chains of MET78, GLY80 and LYS217, which sterically hinders Gaim binding (distance between protruding residues and Gaim<1 Å). Furthermore, a weak electrostatic interaction between LYS217 and ASP411 is predicted to promote a closed pocket formation that further prevents Gaim binding (Fig. 6). Beyond these three pullulanases, *Streptomyces* sp. JHA26 encodes two alpha-1,4-amylases and one cyclomaltodextrin-like amylase. Gaim was predicted to bind all three of these enzymes (data not shown), suggesting that the Gaim occlusion phenotype predicted for WP_077796429.1 (Fig. 6) is unique to this protein within *Streptomyces* sp*. JHA26*.

## Discussion

Streptomycetes are known to inhabit a wide range of ecological niches and exhibit extensive genome plasticity, particularly in their repertoires of BGCs and CAZymes [41]. In this study, it was found that *Streptomyces* species frequently harbour BGCs encoding amylase inhibitors, such as acarbose and tendamistat, alongside inhibitor-resistant amylases. It is known that acarbose not only inhibits amylases but may also function as a ‘carbophor’ for the producing organism [35,42]. In this role, acarbose is covalently linked to glucose or dextrins through the acarviosyltransferase AcbD and then transported into the cytoplasm through the ABC transporter AcbFGH-MsiK [34,35]. Notably, this work indicates that acarbose resistance is more likely achieved through reconfiguration of acarbose binding within the alpha-amylase catalytic pocket rather than exclusion of the inhibitor. This suggests that acarbose-resistant amylases may participate in the carbophor cycle, enabling acarbose-producing organisms to sequester oligosaccharides, which might otherwise be freely accessible to competing organisms, by covalently linking them to acarbose, thereby restricting uptake to organisms possessing the dedicated importer.

This work further reveals substantial diversity in both the number and types of amylases found across the *Streptomyces* genus. It was found that amylases broadly cluster into three major clades that are homologous in their *α*-amylase domains but differ in their adjacent carbohydrate-binding domains. This likely confers substrate specificity towards starches of varying composition, such as those derived from different plant species or fungal sources. Importantly, these conclusions are based on bioinformatic predictions, whereby enzymatic function, substrate specificity and inhibitor interactions were inferred from sequence homology, domain architecture and previously characterized motifs, and remain to be experimentally validated. Despite these limitations, the atlas of amylases presented in this work may provide a useful resource for the targeted selection of enzymes for heterologous overexpression in industrial antibiotic production strains. As such, it may facilitate the optimization of sustainable antibiotic fermentations by deploying amylases with substrate specificities tailored to organic waste streams of different origins. Moreover, this approach may provide a useful source of bacterial strains that produce novel alpha-glucosidase inhibitors for therapeutic use in diabetes mellitus [43].

In the context of engineering starch-fuelled industrial cell factories, it is important to consider the potential repertoire of endogenous amylase inhibitors encoded by a given production strain. Amylase inhibitors are frequently found in *Streptomyces* bacteria, and care must be taken to ensure that any host strain does not produce inhibitors that act against a heterologously expressed amylase, as this could compromise manufacturing efficiency. *Streptomyces hygroscopicus*, for example, is used industrially for the large-scale production of a range of bioactive compounds, including rapamycin [44]. Improving the sustainability of fermentations involving this organism using organic waste substrates, such as bread or potato waste, would be of considerable environmental value [4,45]. However, as this analysis shows, *S. hygroscopicus* (GCF_001652895.1) encodes an acarbose BGC, and this may potentially interfere with amylase activity during fermentation. Strategies to overcome this challenge could be to either delete this cluster or, alternatively, overexpress an acarbose-resistant amylase. The latter approach may be more broadly applicable, as overexpressing genes often is easier than deleting them. Moreover, considering the ongoing antibiotic resistance crisis and the need to discover and industrially deploy new antibiotics, production pipelines should be readily adaptable to newly identified producer strains. Rather than repeatedly facing the challenging task of deleting gene clusters encoding amylase inhibitors in newly characterized strains, it may be more efficient to develop a small set of broadly applicable overexpression vectors. Furthermore, as this and previous work suggest, creating inhibitor-resistant amylases may be achieved through minimal sequence modifications, including single amino acid substitutions. It may hence be possible to develop a toolbox of inhibitor-resistant amylases with optimized substrate specificities towards starches from diverse sources, which could be readily integrated into a wide range of natural product-producing *Streptomyces* strains.

In summary, this work highlights the coordinated evolution of amylases and amylase inhibitors in *Streptomyces*, revealing how starch utilization and inhibitor resistance are tightly integrated. These findings provide insight into how producing organisms balance nutrient acquisition with competitive exclusion and offer a framework for engineering inhibitor-resistant, starch-utilizing strains for sustainable antibiotic fermentations.

## Supplementary material

10.1099/mgen.0.001747 Supplementary Material 1.

## Abbreviations

BGCs: biosynthetic gene clusters
CAZymes: carbohydrate-active enzymes
GH13: glycoside hydrolase category 13
MSA: multiple sequence alignment
ST: sequence type
