# Supplementary material for: Genome mining of amylases and amylase inhibitors from Streptomyces
Source: Microb Genom. 2026 Jun 5;12(6):001747. doi: 10.1099/mgen.0.001747 (PMC13240717; doi:10.1099/mgen.0.001747)
Supplement: Supplementary Material 1. [file mgen-12-01747-s001.pdf]

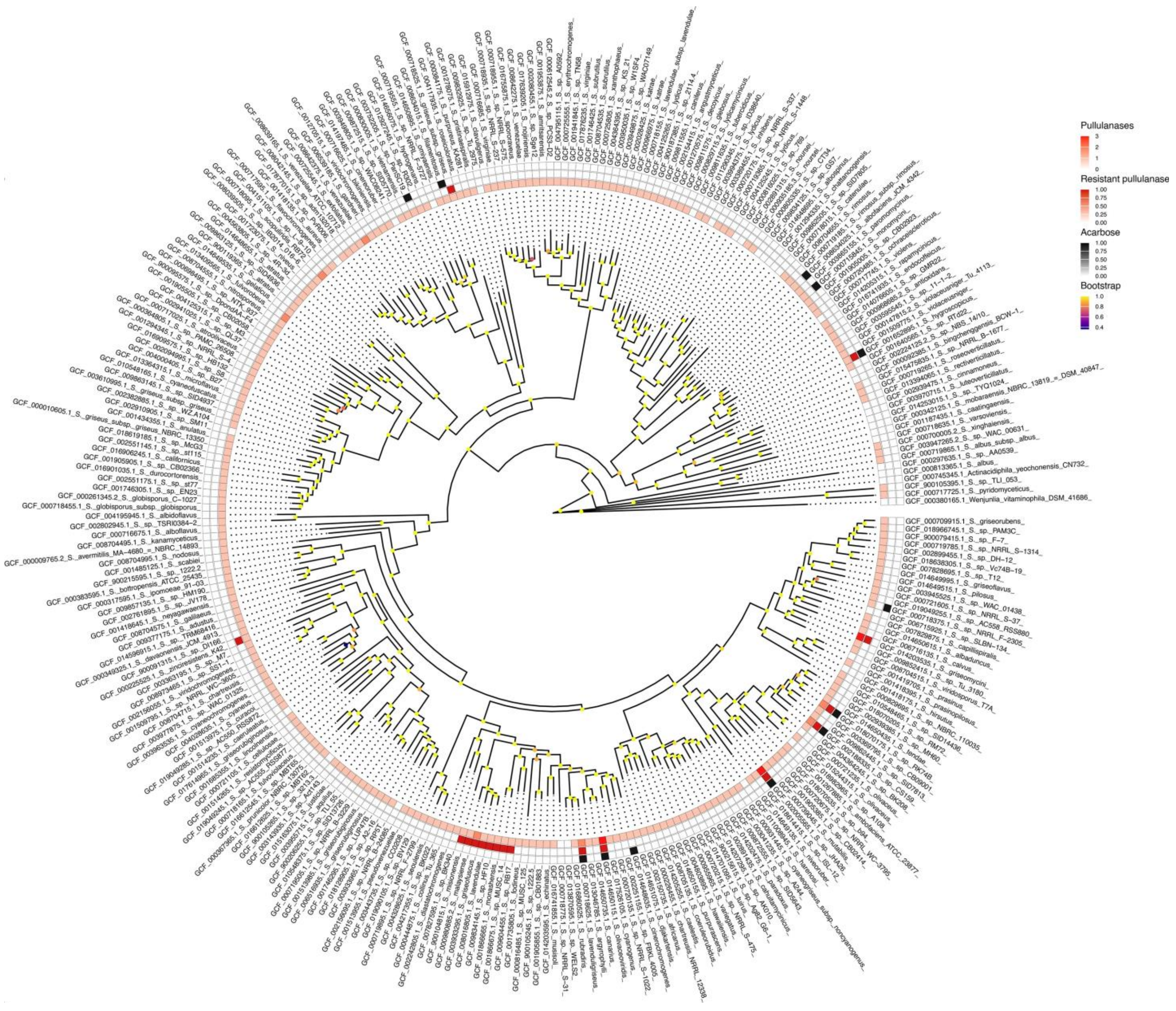


**Supplementary Figure S1: Amylase inhibitor resistant amylases are enriched in genomes encoding acarbose BGCs.**

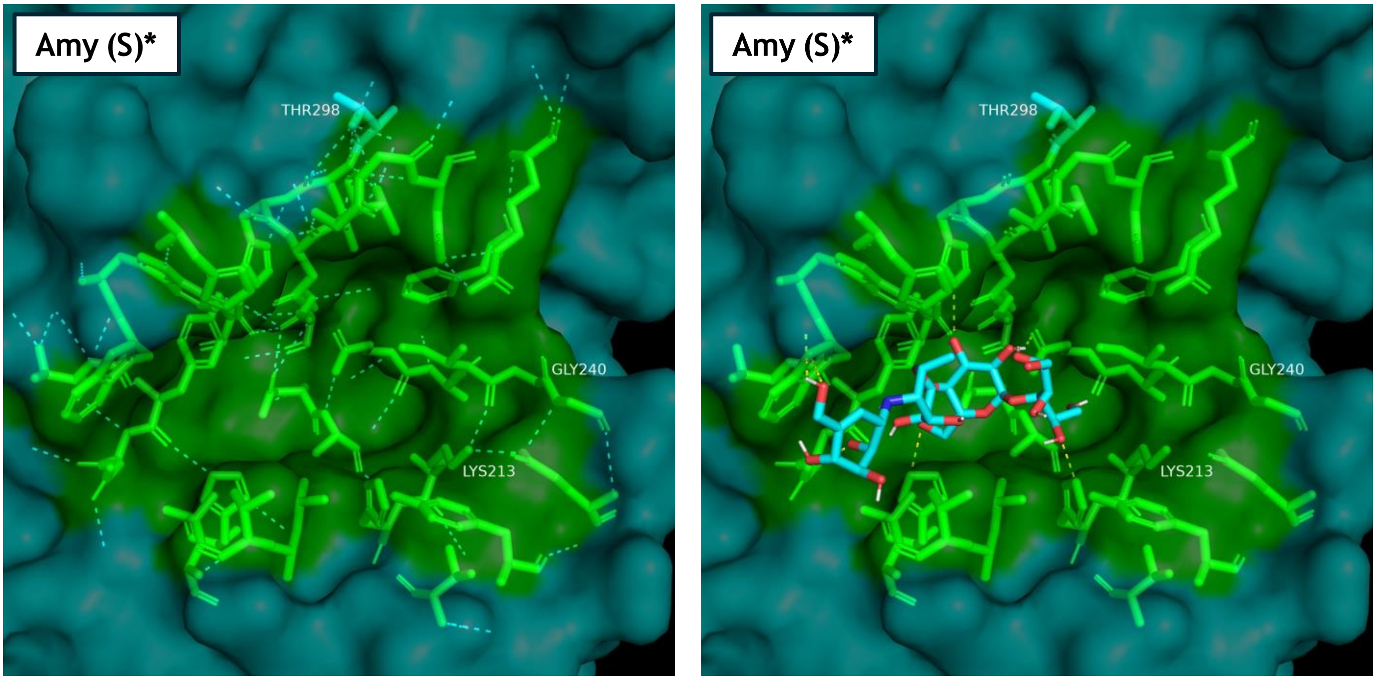


**Supplementary Figure S2: D298T and H240G changes the binding pattern of acarbose in a non-acarbose-associated amylase (WP_103530170) to that seen in acarbose-associated amylases.**

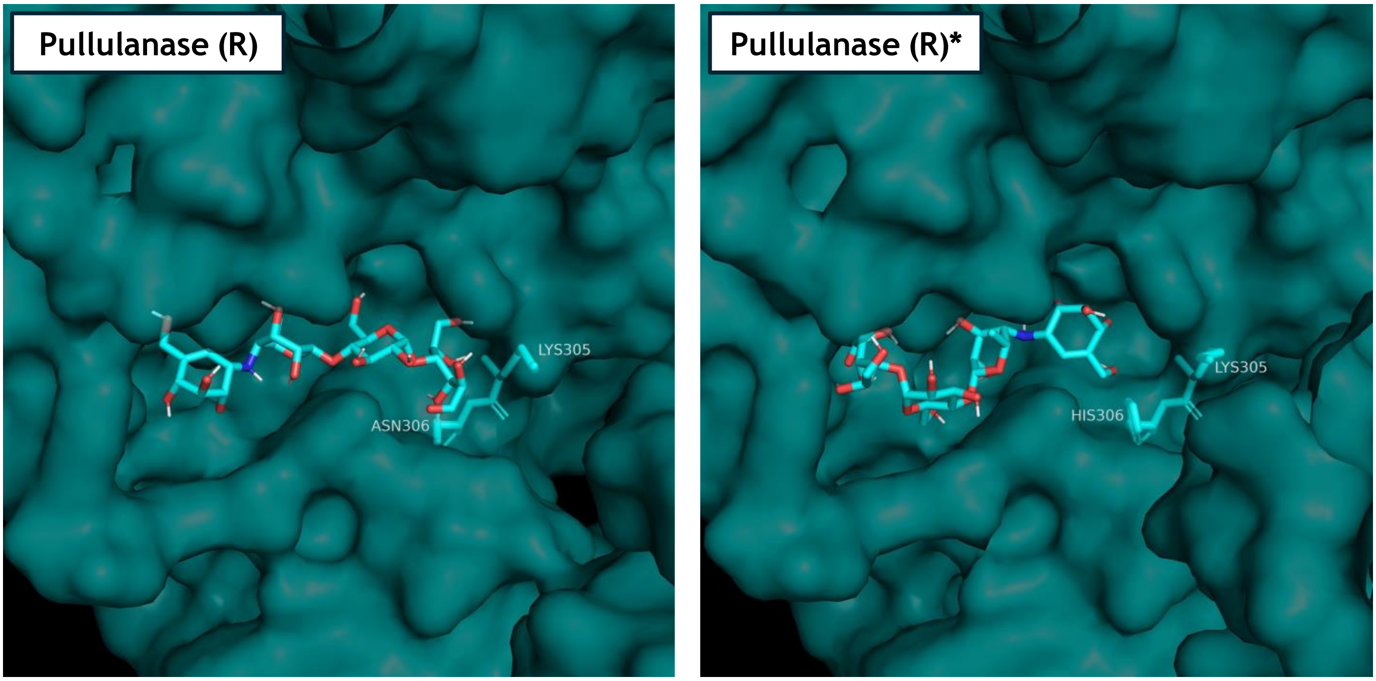


**Supplementary Figure S3: N306H reorients the predicted binding of acarbose in an inhibitor resistant pullulanase (WP_064455463) to a configuration similar to that seen in alpha-1,4-amylases found in genomes without acarbose.**

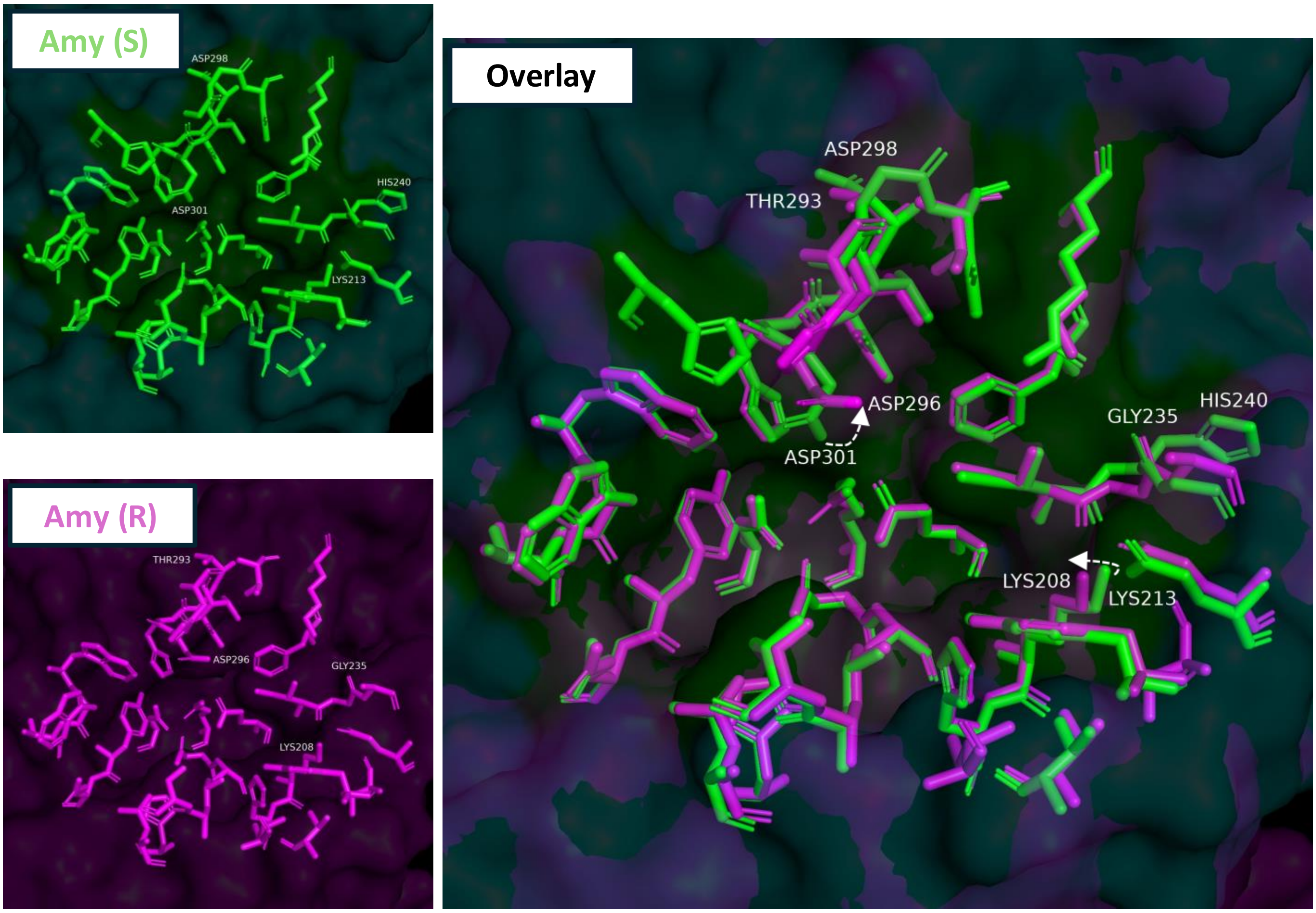


**Supplementary Figure S4: The architecture of the catalytic pocket differs between Amy (S) and Amy (R).** Amy (S) [WP_103530170; green] and Amy (R) [WP_077796428; magenta] varies on residue 293/298 and 235/240 which might be causative of the reorientation of D296/301 and K208/213 resulting in the narrower binding pocket found in Amy (R).

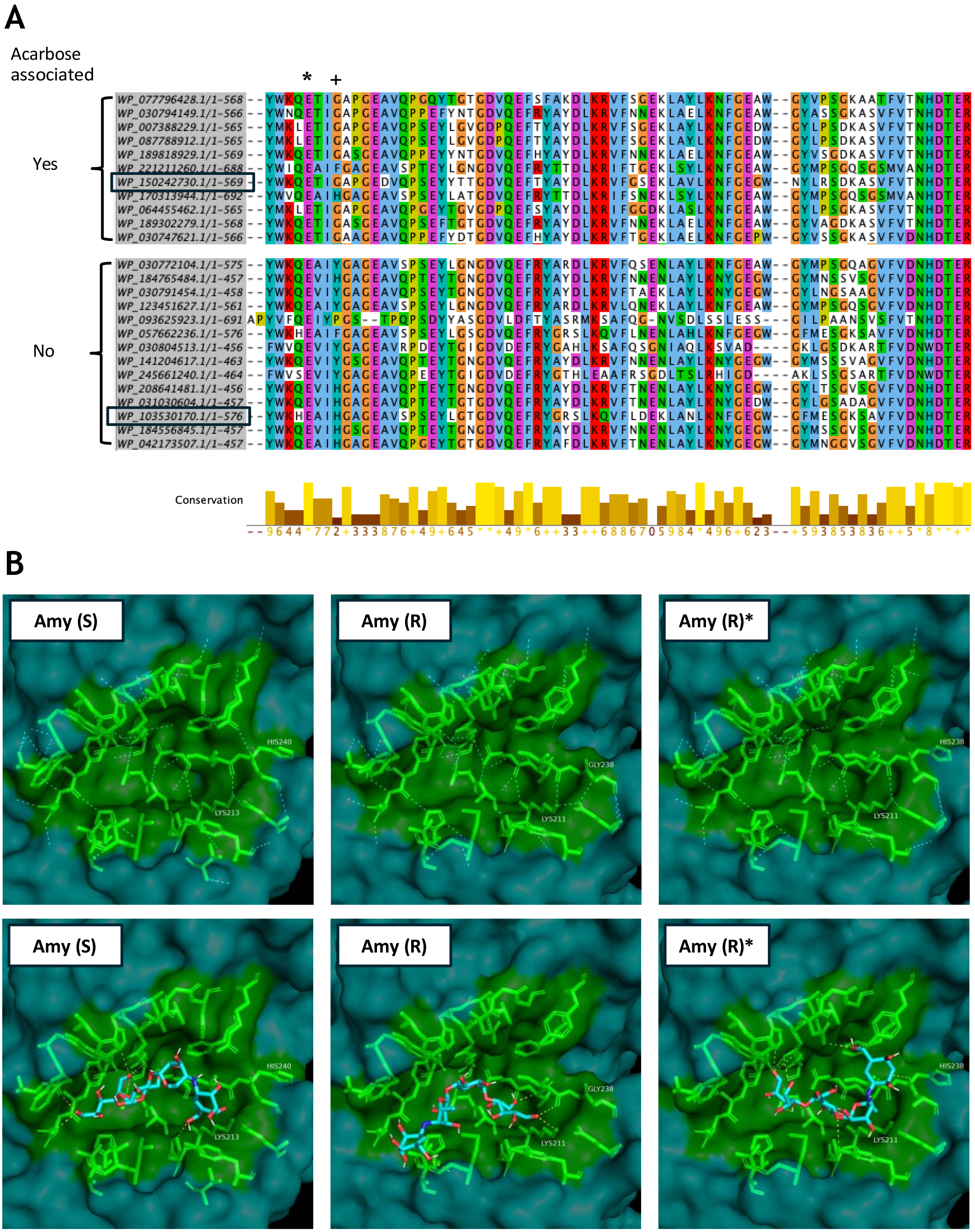


**Supplementary Figure S5: G238H reorients acarbose binding in an $\alpha$-1,4-amylase from an acarbose BGC without an acarbose resistant pullulanase.** MSA shows an enrichment of GLY238 (+) in alpha-1,4-amylases located in acarbose BGCs (A). The catalytic pockets (residues highlighted in green) are shown for a putatively acarbose-sensitive amylase [Amy (S) – accession: WP_103530170] found in a genome without acarbose and a putatively resistant amylase [Amy (R) – accession: WP_150242730] found within an acarbose BGC [proteins marked by black squares in (A)]. Amy (R)* shows the structure of Amy (R) where GLY238 have been converted to HIS, identical to the residue found at this positions in Amy (S). Hydrogen

bonds between residues within the catalytic pocket are shown with dashed cyan lines and hydrogen bonds between acarbose and the proteins are shown with dashed yellow lines (bottom panel). LYS213/211, previously described as involved in inferring acarbose resistance is highlighted (B).
